# A Novel Multi-Dimensional Clinical Response Index Dedicated to Improving Global Assessment of Pain in Patients with Persistent Spinal Pain Syndrome after Spinal Surgery, Based on a Real-Life Prospective Multicentric Study (PREDIBACK) and Machine Learning Techniques

**DOI:** 10.3390/jcm10214910

**Published:** 2021-10-24

**Authors:** Philippe Rigoard, Amine Ounajim, Lisa Goudman, Pierre-Yves Louis, Yousri Slaoui, Manuel Roulaud, Nicolas Naiditch, Bénédicte Bouche, Philippe Page, Bertille Lorgeoux, Sandrine Baron, Elodie Charrier, Laure Poupin, Delphine Rannou, Géraldine Brumauld de Montgazon, Brigitte Roy-Moreau, Nelly Grimaud, Nihel Adjali, Kevin Nivole, Mathilde Many, Romain David, Chantal Wood, Raphael Rigoard, Maarten Moens, Maxime Billot

**Affiliations:** 1 PRISMATICS Lab (Predictive Research in Spine/Neuromodulation Management and Thoracic Innovation/Cardiac Surgery), Poitiers University Hospital, 86021 Poitiers, France; Amine.OUNAJIM@chu-poitiers.fr (A.O.); Manuel.ROULAUD@chu-poitiers.fr (M.R.); nicolas.naiditch@gmail.com (N.N.); dr.bouche@gmail.com (B.B.); Bertille.LORGEOUX@chu-poitiers.fr (B.L.); sandrine.baron@chu-poitiers.fr (S.B.); Nihel.adjali@chu-poitiers.fr (N.A.); Kevin.NIVOLE@chu-poitiers.fr (K.N.); mathilde.many@chu-poitiers.fr (M.M.); romain-david@hotmail.fr (R.D.); chantalwood@orange.fr (C.W.); Maxime.BILLOT@chu-poitiers.fr (M.B.); 2 Department of Spine Surgery & Neuromodulation, Poitiers University Hospital, 86021 Poitiers, France; Philippe.PAGE@chu-poitiers.fr; 3 Pprime Institute UPR 3346, CNRS, ISAE-ENSMA, University of Poitiers, 86360 Chasseneuil-du-Poitou, France; 4 Laboratoire de Mathématiques et Applications UMR 7348, CNRS, University of Poitiers, 86073 Poitiers, France; yousri.slaoui@math.univ-poitiers.fr; 5 Department of Neurosurgery, Universitair Ziekenhuis Brussel, 1090 Brussels, Belgium; lisa.goudman@gmail.com (L.G.); mtmoens@gmail.com (M.M.); 6 STIMULUS Research Group, Vrije Universiteit Brussel, 1090 Brussels, Belgium; 7 AgroSup Dijon, PAM UMR 02.102, Université Bourgogne Franche-Comté, 21000 Dijon, France; pierre-yves.louis@agrosupdijon.fr; 8 Institut de Mathématiques de Bourgogne, UMR 5584 CNRS, Université Bourgogne Franche-Comté, 21000 Dijon, France; 9 Pain Evaluation and Treatment Centre, Poitiers University Hospital, 86021 Poitiers, France; Elodie.CHARRIER@chu-poitiers.fr (E.C.); laure.poupin@chu-poitiers.fr (L.P.); delphine.rannou@chu-poitiers.fr (D.R.); 10 Pain Evaluation and Treatment Centre, La Rochelle Hospital, 17000 La Rochelle, France; Geraldine.DEMONTGAZON@ght-atlantique17.fr; 11 Pain Evaluation and Treatment Centre, Nord Deux-Sèvres Hospital, 79000 Niort, France; Roy-Moreau.Brigitte@chnds.fr; 12 Pain Evaluation and Treatment Centre, Centre Clinical Elsan, 16800 Soyaux, France; ngrimaud@centre-clinical.fr; 13 Physical and Rehabilitation Medicine Unit, Poitiers University Hospital, University of Poitiers, 86021 Poitiers, France; 14 CEA Cadarache, Département de Support Technique et Gestion, Service des Technologies de l’Information et de la Communication, 13108 Saint-Paul-Lez-Durance, France; cydran@gmail.com

**Keywords:** composite score, machine learning, PSPS, failed back surgery syndrome (FBSS), chronic pain, pain intensity, quality of life, pain mapping, pain surface, functional capacity, psychological distress, anxiety and depression

## Abstract

The multidimensionality of chronic pain forces us to look beyond isolated assessment such as pain intensity, which does not consider multiple key parameters, particularly in post-operative Persistent Spinal Pain Syndrome (PSPS-T2) patients. Our ambition was to produce a novel Multi-dimensional Clinical Response Index (MCRI), including not only pain intensity but also functional capacity, anxiety-depression, quality of life and quantitative pain mapping, the objective being to achieve instantaneous assessment using machine learning techniques. Two hundred PSPS-T2 patients were enrolled in the real-life observational prospective PREDIBACK study with 12-month follow-up and received various treatments. From a multitude of questionnaires/scores, specific items were combined, as exploratory factor analyses helped to create a single composite MCRI; using pairwise correlations between measurements, it appeared to more accurately represent all pain dimensions than any previous classical score. It represented the best compromise among all existing indexes, showing the highest sensitivity/specificity related to Patient Global Impression of Change (PGIC). Novel composite indexes could help to refine pain assessment by informing the physician’s perception of patient condition on the basis of objective and holistic metrics, and also by providing new insights regarding therapy efficacy/patient outcome assessments, before ultimately being adapted to other pathologies.

## 1. Introduction

A substantial fraction of spine surgery patients (10–50%) [1] develop new or persistent back and/or leg pain postoperatively [2,3,4,5]. Previously known as failed back surgery syndrome, this pain entity was recently classified as persistent spinal pain syndrome type 2 (PSPS-T2) [6,7]. As with other types of pain, PSPS-T2 is viewed as an unpleasant sensory and emotional experience influenced by biological, psychological, and social factors, leading to a decrease in health-related quality of life (QoL) [7,8,9,10]. PSPS-T2 constitutes a major public health issue and financial burden for society [11]. The heterogeneity of PSPS-T2 etiologies [12,13] and patient characteristics [14,15] makes it difficult to identify with clarity which therapeutical option should be prioritized, the objective being to obtain the best outcomes for patients with varied and complex care pathways.

Despite constantly innovative digital technology and Artificial Intelligence (AI), pain is still assessed by “gold-standard tools” such as the Numerical Pain Rating Scale (NRPS) score, which ranges from 0 (no pain) to 10 (the worst imaginable pain), [16]. Massively influencing daily pain practice (as an example, change in opioid prescription is traditionally based on NPRS ≥ 4), these numerical scales serve as reference cut-offs for eligibility to therapies. For example, a pain decrease ≥50% on the VAS in a chronic refractory patient must be observed before considering a Spinal Cord Stimulation (SCS) trial successful and proceeding to a permanent SCS implant [17,18]. The main advantage of the scales is ease of use, while their major limitation is to fail to consider more than one of the many dimensions of pain, such as functional disability [10,19,20,21] or psychological distress [2,10,22].

Other limitations of unidimensional sporadic pain intensity assessment scales consist in their being unable to quantitatively capture positional changes, multifocal pain, mixed pain components [23,24], daily variability of pain depending on efforts, mechanical loads, pain typology, pain characterization, influence of psycho-social factors and impact on function. Often catalyzed by the side effects of pain medication, these different components are not seen, as pieces of a single complex puzzle, colonizing and progressively devastating all dimensions of a chronic pain patient’s life. 

In daily practice, pain intensity, functional disability, psychological distress and quality of life assessments are considered subjectively and independently, even though clinical experience underscores the massive interlaying of these heterogeneous but permanently interconnected, pain dimensions. 

In view of better understanding PSPS-T2 patient profiles, mixed-effect regression models have been used to determine the impact of pain intensity, functional disability, and psychological distress on health-related Quality of Life (QoL) perception [10]. In this study, Ounajim et al. [10] showed that 2 classes of PSPS-T2 patients can be identified; while the first corresponds to those whose QoL is mainly affected by functional disability and psychological distress, the second class corresponds to those whose QoL is mainly affected by pain intensity as well as psychological distress. With robust evidence, this study showed that changes in pain intensity over time fail to reflect the evolution of a chronic pain patient’s QoL. On the contrary, a multidimensional composite score should reflect a holistic evaluation [25], and potentially provide a reliable standardized clinical assessment of therapy efficacy, in the context of a particularly complex care pathway. 

In alignment with this approach, in a real-life observational prospective study we have introduced machine learning techniques leading to the creation of a Multidimensional Clinical Response Index (MCRI) representing with high accuracy the global health status of PSPS-T2 patients. Our objectives were (i) to determine whether, as a single composite index, the MCRI would reflect all/each of the pain dimensions more accurately than any other available pain score and, (ii) to compare the sensitivity/specificity of each existing pain score vs. MCRI, especially as regards correlation with Patient Global Impression of Change (PGIC).

## 2. Materials and Methods

### 2.1. Study Design

This prospective observational multicenter study, called PREDIBACK study, was designed to develop a new Multidimensional Clinical Response Index (MCRI). The study is a multivariate research design where several outcomes (ODI, EQ-5D, HADS, NPRS and pain surface) were combined to create the multidimensional outcome MCRI, and a correlational study evaluating the strength of association between the different outcomes.

The study protocol was registered on Clinicaltrial.gov as NCT02964130 on 15 November 2016. The study was approved by the ANSM (2016-A01144-47) as well as by the Ethics Committee West III and complied with the Declaration of Helsinki. Participants received explanations of the study and provided written informed consent before enrolment in this study.

### 2.2. Participants

#### 2.2.1. Inclusion Criteria

Recruitment of 200 PSPS-T2 patients was conducted in 5 French pain centers (Angoulême, Bressuire, La Rochelle, Niort and Poitiers) from January 2017 to Mars 2018. The patient eligibility was determined at each site through standard clinical practice and all patients provided consent before enrolment. They had to be older than 17 years; to have undergone most recent back surgery more than 6 months before; to be suffering persistent back and/or leg pain after spinal surgery for more than 6 months; and to have an average pain score ≥ 4/10 on the Numeric Pain Rating Scale (NPRS).

#### 2.2.2. Exclusion Criteria

Excluded from the study were patients with history of past or current treatment with spinal cord, subcutaneous or peripheral nerve stimulation, with an intrathecal drug delivery system; had a previously confirmed PSPS-T2 diagnosis; had life expectancy of less than 12 months after study enrolment; were unable to undergo study assessments or complete questionnaires independently; were a member of a vulnerable population; and/or the investigator suspected substance abuse that might confound the study results.

### 2.3. Objectives

Our primary objective was to develop a Multidimensional Clinical Response Index (MCRI) reflecting multidimensional pain assessment in a population of 200 patients presenting with PSPS-T2 and receiving various treatments during 12 months of follow-up. The secondary objectives were to determine (i) the correlation between MCRI and pain intensity score, quality of life, functional capacity score, anxiety/depression score, and pain mapping intensity changes, (ii) the comparative predictive power of the MCRI to detect and reflect clinical changes in pain intensity, quality of life, functional capacity, anxiety/depression and pain mappings, based on Patient Global Impression of Change (PGIC) at 3 (M3), 6 (M6), and 12 (M12) month follow-up.

### 2.4. The Multidimensional Clinical Response Index (MCRI) 

#### 2.4.1. Input Data

To develop the MCRI, pain intensity, quality of life, functional capacity, anxiety/depression, pain mapping measurements were collected at baseline. 

Pain intensity was measured by means of a Numerical Pain Rating Scale [26] (NPRS), ranging from 0 (no pain) to 10 (maximal pain that they could imagine). Clinical effectiveness was assessed in terms of quality of life (EuroQol 5-Dimensions 5-Level questionnaire (EQ-5D-5L)) [27], functional disability (The Oswestry Disability Index questionnaire (ODI)) [28], anxiety/depression (The Hospital Anxiety and Depression Scale (HADS)) [29], Pain Mapping Intensity (PMI) changes (pain surface according to the pain intensity, PRISMap software) [30]. PRISMap accurately localizes the pain surface (in cm²) associated with a coefficient related to pain intensity. Patients draw their pain surface directly on a computerized tactile interface in a predetermined body (individually adapted from the patient body mass index). A color code was used to signify pain intensity: red = very intense, orange = intense, dark blue = moderate, light blue = mild [30]. Lower pain intensity was associated with coefficient 1, medium pain intensity with coefficient 2, intense pain intensity with coefficient 3, and very intense pain intensity with coefficient 4. 

The equation was written as:

PMI = 1 * SurfaceLow + 2 * SurfaceMedium + 3 * SurfaceIntense + 4 * SurfaceVeryIntense.

Surface is the pain surface in cm^2^, calculated by patented processing, associated with intensity ranging from 1 to 4, where 1 is low pain, 2 is medium pain, 3 is intense pain and 4 is very intense pain.

#### 2.4.2. Variable Reduction and Factor Analysis

Correlation between items was determined using the repeated measure correlation coefficient. Cronbach alphas were calculated at baseline for each questionnaire to assess the internal validity of the EQ-5D-L, ODI, HADS anxiety and depression subscales. The 29 items from the 3 questionnaires (i.e., EQ-5D-5L, ODI and HADS) were gathered together in a single questionnaire. Global NPRS and PMI were retained as scores representing the “pain intensity” and “pain surface intensity” constructs. 

#### 2.4.3. Reduction of the Number of Items

A subset of items from each questionnaire (EQ-5D-5L, ODI, HADS) were selected and used to determine the final dimensions of the MCRI.

First, based on the construct they measured, clinically redundant items were deleted from the questionnaires. Second, an Exploratory Factor Analysis (EFA) was conducted in order to remove items with very low loadings (i.e., loading < 0.3) in all the factors. We also removed items with very high loadings on the same factor. Highly correlated, these items were producing redundant information. The item with the higher loading between two redundant items was retained. The Kaiser–Meyer–Olkin measure of sampling adequacy was used to assess the suitability of our data for factor analysis. In the EFA step, the number of latent factors was determined using the Very Simple Structure (VSS) criterion, parallel analysis [31] and theoretical validity (the clinical relevance factor). For factor extraction, we used the principal axis factoring method. Promax rotation provided correlated factors (called oblique solutions) insofar as the different chronic pain dimensions (i.e., pain intensity and functional disability) were correlated.

A confirmatory factor analysis was conducted to test whether the factors obtained represented an underlying construct of the items they contained. To account for the clustered nature of the data (repeated measures longitudinal data) we used a two-level CFA (within-patient and between-patient effects). In CFA, model parameters were estimated using the maximum likelihood estimation method with robust (Huber–White) standard errors and a scaled test statistic [32] where all the variables were standardized to allow comparability. As our variables did not have normal multivariate distribution (tested using Henze–Zirkler’s multivariate normality test), model fit was tested using a Chi-squared test with Yuan–Bentler correction. Goodness of fit was assessed using the Root Mean Square Error of Approximation (RMSEA) and the Comparative Fit Index (CFI). An RMSEA value under 0.05 indicates an excellent fit, while values between 0.05 and 0.08 indicate an acceptable fit. For CFI, a value between 0.90 and 0.95 is considered acceptable and a value of 0.95 or greater indicates an excellent fit.

EFA was conducted using the psych package while CFA was conducted using the lavaan package on R software (Version 3.6.0; R Foundation for Statistical Computing, Vienna, Austria).

#### 2.4.4. Final Factor Analysis and Unidimensional Assessment

The previously developed EFA results (factors and their item loadings) were used to determine each factor score. Factor scores were extracted through the Bartlett approach [33].

Patients were described by each of the pain constructs represented by the scores extracted from the EFA plus pain intensity (NPRS score) and PMI. Each constructed score was standardized to a [0,100] interval to improve interpretability. Baseline correlation of the scores was obtained and the standard pain evaluation measures (i.e., NPRS, ODI score, EQ-5D index, HADS total score and PMI) were determined using Spearman’s rho and its 95% confidence interval.

#### 2.4.5. Multidimensional Assessment

Scores from the different constructs were included in a Principal Component Analysis (PCA) in order to obtain a new assessment score summarizing the patient’s pain state. The first component of the PCA was used as a summary score named MCRI, which was standardized to [0,10] to facilitate interpretation. We also used PCA on the original scores of the questionnaires (i.e., ODI percentage, HADS total score, NRPS, EQ-5D index and PMI) for purposes of comparison with the MCRI developed by EFA.

#### 2.4.6. Correlation between MCRI and NPRS, EQ-5D, ODI, HADS and PMI

NRPS, ODI, EQ-5D, NPRS, HADS, and PMI were collected at 3, 6, 9 and 12-month follow-up. The MCRI was then calculated for the M3, M6 and M12 follow-up periods. The correlation between MCRI and the other parameters was calculated from M3 to M12 using Spearman rho coefficients.

#### 2.4.7. Identifying the Patient Global Impression of Change (PGIC) Using the MCRI, NPRS, EQ-5D, ODI, HADS and PMI

Patient satisfaction was assessed with Patient Global Impression of Change (PGIC). PGIC is a 7-point scale depicting a patient’s rating of overall improvement from 0 (very much worse” to 7 (very much improved) [34]. Self-perceived clinical improvement is considered satisfactory when the patient reports a PGIC score ≥ 6 and is considered unsatisfactory when the patient reports a PGIC score ≤ 5. The Area Under the ROC Curve (AUC) for detection of satisfactory self-perceived clinical improvement (PGIC score ≥ 6) was calculated using the change between baseline and at 3, 6, 9 and 12-month follow-up for all outcomes (MCRI, ODI, EQ-5D, NPRS, PMI and HADS). We also calculated the optimal cut-off points of the changes in the MCRI, NPRS, EQ-5D, ODI, HADS, and PMI based on simultaneous maximization of specificity and sensitivity in the detection of satisfactory self-perceived clinical improvement.

The relationship between PGIC and the change score was tested using the Jonckheere–Terpstra test, which presupposes a trend (increase or decrease) in the distribution location statistic between the ordinal variable groups. A *p*-value of less than 0.05 was considered statistically significant.

Missing values were not imputed; data were analyzed according to an available-case principle.

## 3. Results

### 3.1. Follow-Up and Missing Data Description

**Figure 1:**
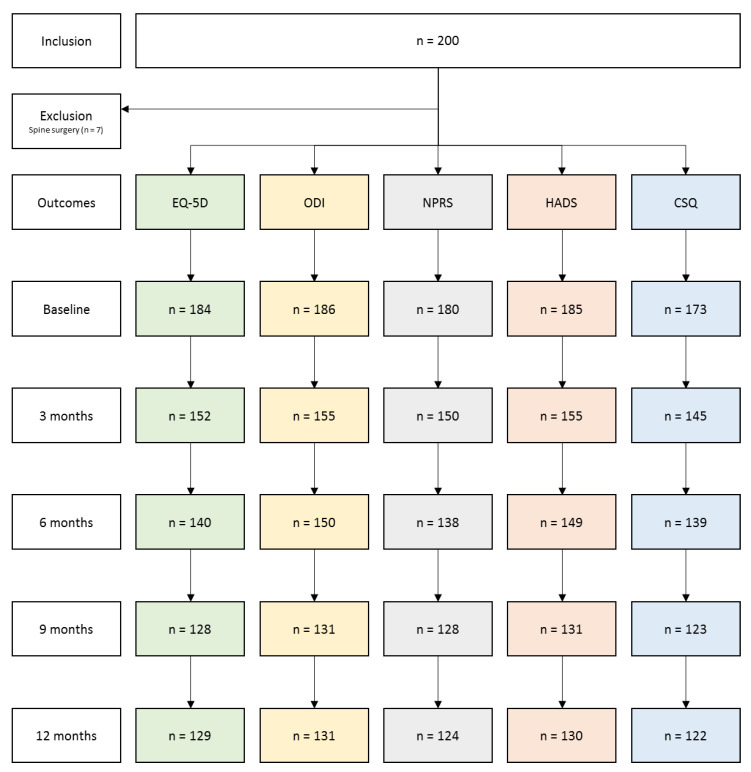
Flowchart of study participants. EQ-5D: EuroQol-5 Dimensions; ODI: Oswestry Disability Index; NPRS: Numeric Pain Rating Scale; HADS: Hospital Anxiety and Depression Scale; CSQ: Coping Strategies Questionnaire.

Out of the 200 included patients, 7 were removed from the analysis due to spine surgery (flowchart in Figure 1). Out of the remaining 193 patients, 186 (96.4%) completed questionnaires at baseline, 155 (80.3%) at 3-month, 150 (77.7%) at 6-month and 131 (67.9%) at 9- and 12-month follow-up.

### 3.2. Descriptive Statistics of Study Sample at Baseline

At baseline, the study participants’ mean age was 52.9 ± 12.5 years, 110 (57.0%) were females and 83 (43.0%) were males. Ninety-eight patients (50.8%) had undergone at least two spinal surgeries, 55 patients (28.5%) had undergone at least three spinal surgeries, and the 40 remaining patients (20.7%) had undergone four or more spinal surgeries. At baseline, the EQ-5D score was 0.28 ± 0.23, the global NPRS was 6.1 ± 1.5, the ODI percentage was 44.6 ± 13.2%, and the HAD depression score was 8.6 ± 3.9.

### 3.3. Variable Reduction and Factor Analysis

Due to their redundancy, 3 items were removed from the questionnaire: 2 items from the EQ-5D questionnaire (item-4 “Anxiety/Depression” and item-5 “Pain/Discomfort”) and 1 item from the ODI (item-1 “Pain intensity”).

#### 3.3.1. First Exploratory Analysis

The first exploratory analysis was performed on the 26 remaining items using data from all time points. This initial EFA showed a 2-factor structure, which explained 22% of total variance. The Kaiser–Meyer–Olkin measure of sampling adequacy was 0.87, indicating adequacy of the sample. The first factor consisted of 9 items out of 10 from the ODI questionnaire, 3 items out of 5 from the EQ-5D-5L, and 1 item out of 14 from the HAD. The second factor consisted in the remaining items of the HADS questionnaire (13 items), where 3 items had loadings < 0.3. These 3 items were consequently removed from the analysis.

#### 3.3.2. Final Exploratory Analysis

**Figure 2:**
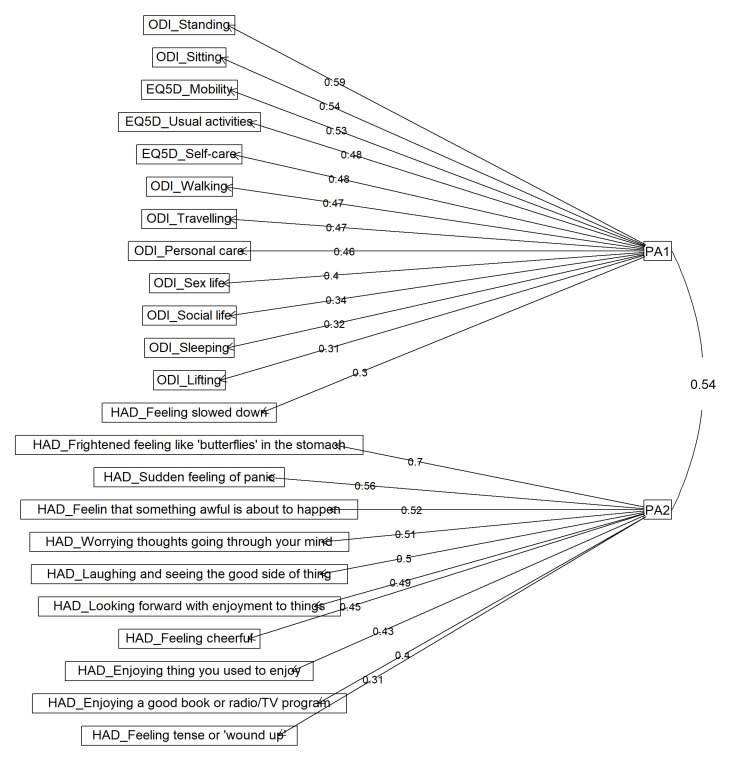
The EFA results with a promax rotation and principal axis factor extraction. (PA1) is the factor associated with functional disability and PA2 is the factor associated with depression/anxiety (PA2). EQ-5D: EuroQol-5 Dimensions; ODI: Oswestry Disability Index; HAD: Hospital Anxiety and Depression.

A final exploratory factor analysis with 2 factors was performed on the 23 remaining items. The Kaiser-Meyer-Olkin measure of sampling adequacy was 0.87, indicating that a factor analysis was suitable for all. In a parallel analysis, we found that the eigenvalues from the current data were greater than those of the simulated random data for 2 factors. The VSS criterion also supported the two-factor structure. The factor loadings obtained this exploratory factor analysis and the correlations between these factors are presented in Table 1. Figure 2 presents the structure of the final 2-factor model and Table 1 the items represented in the functional disability (PA1) and depression/anxiety (PA2) factors.

#### 3.3.3. Confirmatory Factor Analysis and Item Selection

The results of the EFA (23 items and 2 factors) were used to construct our CFA model. Details of the standardized coefficients, 95% confidence intervals and *p*-values for within and between-patient effects of the CFA model are presented in Table 2. All coefficients were significant in their respective factors, except for the “Enjoying a good book or radio/TV program” item of the “depression and anxiety” factor.

The goodness of fit model was inconclusive (Table 3). While our CFI model indicated poor fit (0.848 < 0.9), the RMSEA (0.046, 90% CI = [0.042,0.050] *p*-value (H0: RMSEA ≤ 0.05) = 0.9) indicated a good fit. The Chi-squared test with Yuan–Bentler correction was significant (*p* < 0.001), indicating poor fit.

The goodness of fit of each factor was examined using a one-factor CFA including only the items associated with the factor (Table 3). The “depression/anxiety” factor was found to have the lowest goodness of fit measures (CFI = 0.886; RMSEA = 0.064). 

#### 3.3.4. Unidimensional Assessment

**Figure 3:**
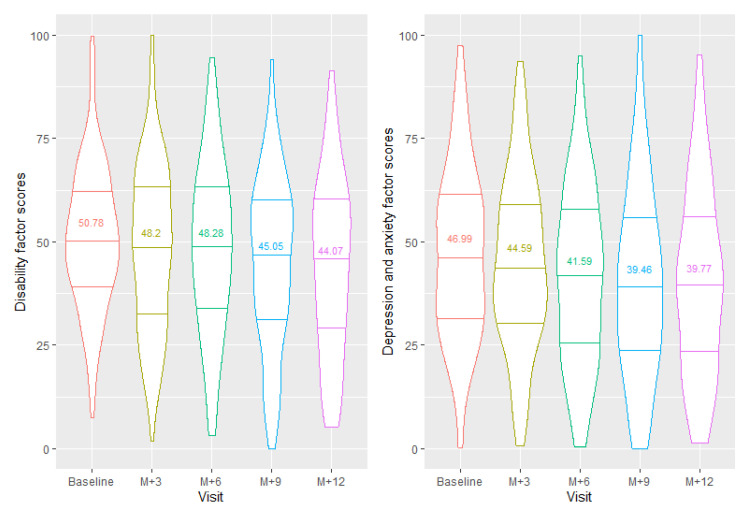
Violin plot of factor scores per visit of each construct. The lines represent the median and the first and third quartiles. The mean scores (/100) of each visit were also added to the plot. The violin plots show the distribution shape of the data at each visit. Wider sections of the violin plot represent a higher probability that patients will have the given score; thinner sections represent a lower probability.

The Bartlett method was used to extract each factor score from the final EFA. Figure 3 presents the distribution of the scores obtained from our EFA (scaled to [0,100]). Table 4 presents the correlations between the scores obtained and the ODI score, EQ-5D index, NPRS and HADS total score at baseline. More specifically, our results showed that the “depression & anxiety” factor was highly correlated with the HADS total score, although the number of items in this factor was smaller than the entire HADS questionnaire (10 out of 14 items) (rho = 0.96; 95% CI = [0.94,0.97]; *p* < 0.0001). Similarly, the “functional disability” factor was highly correlated with the ODI score (rho = 0.92; 95% CI = [0.89,0.94]; *p* < 0.0001) and the EQ-5D index (rho = −0.81; 95% CI = [−0.86,−0.74]; *p* < 0.0001).

### 3.4. Multidimensional Assessment 

Principal Component Analysis (PCA) including the scores extracted from the EFA model, the NPRS scores and the PMI was used to determine the MCRI. First principal component loadings, percentage of explained variance and first eigenvalue are presented in Table 5. The first component of the PCA explained 49.99% of the total variance. All variables had significant loadings in the PCA (>0.3). Loading was −0.811 for functional disability, −0.771 for NRPS, −0.684 for depression & anxiety score and −0.529 for PMI score. 

We scaled the MCRI from 0, indicating the worst pain-related health status, to 10, indicating the best pain-related health status.

### 3.5. The Correlation between MCRI and NPRS, EQ-5D, ODI, HADS and PMI 

**Figure 4:**
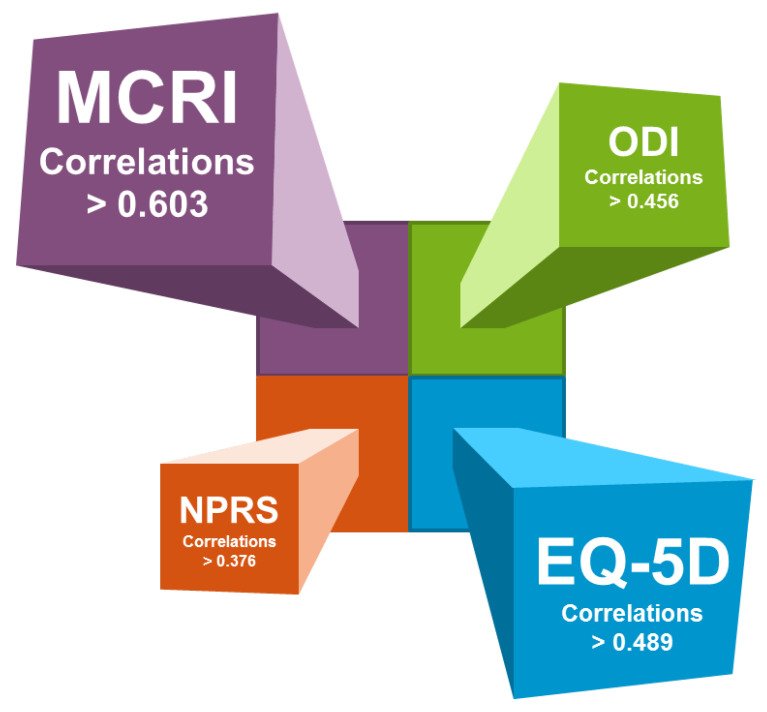
An illustrative representation of the top 4 correlations reflecting pain dimensions.

The correlation between MCRI and the EQ-5D, ODI, NPRS, total HADS and PMI at baseline and at 3-, 6-, 9- and 12-month follow-up are presented in Table 6 and Figure 4. 

The MCRI score was significantly correlated with the ODI score (rho = −0.677; 95% CI = [−0.748,−0.591]; *p*-value < 0.0001), the EQ-5D score (rho = 0.690; 95% CI = [0.606,0.759]; *p*-value < 0.0001) and the NPRS score (rho = −0.677; 95% CI = [−0.749,−0.589]; *p*-value < 0.0001) at baseline. The correlations of the MCRI with the HADS score and PMI were moderate (HADS: rho = −0.622; 95% CI = [−0.703,−0.525]; *p*-value < 0.0001; PMI: rho = −0.423; 95% CI = [−0.533,−0.299]; *p*-value < 0.0001). The correlations at 3-, 6-, 9- and 12-month follow-up were also statistically significant and ranged from −0.527 to −0.828 (Table 6). All correlations were greater at 12 months than at baseline.

Considering pairwise correlations between scores from EQ-5D, ODI, NRPS, HADS and PMI, we found that all the correlations were lower than those obtained with the MCRI (Table 6).

### 3.6. Identification of the Patient Global Impression of Change (PGIC) Using the MCRI, NPRS, EQ-5D, ODI, HADS and PMI 

Out of the 125 patients who reported their PGIC score at 12-months, 31 (24.8%) had satisfactory self-perceived clinical improvement (≥6) and 94 (75.2%) had unsatisfactory self-perceived change (≤5).

Table 7 presents the specificity and sensitivity of the MCRI, ODI, EQ-5D, NPRS and total HADS changes in detection of satisfactory and unsatisfactory self-perceived clinical improvement from the PGIC. The Jonckheere–Terpstra test showed a significant relationship between the change in the MCRI score and the PGIC from baseline to 12-month follow-up (*p* < 0.0001). Likewise, the PGIC was associated with the change in ODI, NPRS, EQ-5D, HADS and PMI scores (*p* < 0.0001). 

The AUC showed that changes in MCRI were the indicator most accurately identifying satisfactory self-perceived clinical improvement (AUC = 0.853) in comparison with the changes of the HADS total score (AUC = 0.780), ODI score (AUC = 0.737), NPRS (AUC = 0.704), EQ-5D index (AUC = 0.698) and PMI score (AUC = 0.672). 

## 4. Discussion

Based not only on pain intensity but also on quality of life, functional disability, anxiety/depression, quantitative pain surface and intensity change assessments, we designed a multiplexed approach applying machine learning methods to capture the essence of pain with an alternative vision. We developed a novel Multidimensional Clinical Response Index (MCRI) to improve global assessment in patients with Persistent Spinal Pain Syndrome after spinal surgery. Compared to other available indexes/scores, MCRI appears to be more robust when considering (i) pairwise correlations between each measurement and (ii) the sensitivity and specificity related to the patient global impression of change (PGIC). The findings of this study accurately define PSPS-T2 patient profiles with a global composite score and could be applied to analyze these patients’ therapeutic pathways.

### 4.1. The NRPS Score: A Gold-Standard Tool Designed to Assess Patient Pain and to Conduct Research on Pain, and Also a Uni-Dimensional Subjective Reflection of a Complex Puzzle. Past and Future Considerations

Nowadays, pain evaluation is systematically and primarily assessed with subjective tools such as the Numerical Pain Rating Scale (NRPS), Visual Analog Scale (VAS), Brief Pain Inventory (BPI), Likert scale, etc. While necessary, these scales provide general and descriptive information that strongly limits the accurate characterization needed to treat chronic neuropathic pain patients, especially PSPS-T2 patients. First, while subjective scales are demonstrably applicable to acute pain at an instant “t”, they may neglect to take into account inter-individual variability. Secondly, these tools alone are not able to differentiate the mechanical from the neuropathic components of pain in a given individual. PainDetect [35,36] and DN4 [37] questionnaires have been used in routine practice to bridge this gap. However, the aforementioned scales are essentially dedicated to determining the relative value of pain changes over time and/or following a treatment application, and are not designed to provide a global picture of health-related quality of life. Another main limit is that while such tools provide global scores for a given individual, pain can affect a variable pain area or even be multifocal. Even though previous studies have used paper map drawings to determine pain location [38], they have failed to offer objective measurements. In our study, on the other hand, pain localization was performed with patented processing encapsulated in a software application (PRISMap), enabling objective quantification of the pain surface changes in cm² [30]. The functional impact of pain will differ for patients whose pain is localized at the upper limb extremities compared to patients with pain localized at the lower limb extremities.

As chronic pain involves multidimensional components, IMMPACT guidelines for pain assessment recommend inclusion if 1 or more measurements of pain, as well as mean changes in physical and emotional functioning [39,40]. Following these recommendations, we used 2 measurements to assess pain (NRPS and PMI), one for physical function (ODI) and the other for the psychological component (HADS). Previous research characterizing 163 PSPS-T2 patients showed that health-related quality of life was affected by several components [10]. Using a mixture model approach, the authors showed that two classes of PSPS-T2 patients can be determined on the basis of three dimensions: pain intensity, functional disability and psychological distress. The ‘pain intensity’ class comprised patients for whom health-related quality of life was more impacted by pain intensity and psychological distress, while the ‘functional disability’ class comprised patients for whom health-related quality of life was more impacted by functional disability and psychological distress. While psychological distress has been considered as systematically impacting health-related quality of life, one-third of the PSPS-T2 patients were assigned to the ‘pain intensity’ class and two-thirds to the ‘functional disability class’. These findings corroborate those of Ballantyne and Sullivan [41,42], who claimed that in attempts to achieve chronic pain relief, the systematic targeting of pain intensity should not be primary. Taken together, these conclusions support the multidisciplinary approach provided by the biopsychosocial model [43,44].

### 4.2. PSPS-T2 Patient Pathway

For the Physician, a Ridgeline in the Devastated Landscape of Pain. For the Patient an Everest to Climb. Focusing only on pain intensity or functional capacity represents an overly narrow viewpoint. In a broader perspective, low education level, lack of adaptive coping strategies and higher pain intensity were found to be significantly associated with HRQoL and more impacted by pain perception [10]. By contrast, males perceiving their work as physical were more impacted by disability than pain intensity [10]. Corroborating these findings, Naiditch et al. [9] reported that low Social Gradient of Health (SGH), a concept used to elucidate the relationship between socioeconomic position and health, was overrepresented in PSPS-T2 patients (85.3%) as compared to the general population (62.8%). PSPS-T2 patients with low SGH also presented significantly higher kinesiophobia, catastrophizing, and functional disability scores than their high SGH counterparts. Proposing “Adapted Professional Activity” as a possible mirror image of Adapted Physical Activity, another study reported that inactive patients were more likely than active patients to develop PSPS-T2 syndrome, especially when their profile was associated with low SGH [45]. The authors proposed a specific PSPS-T2 patient pathway, with initial clinical assessment including patient clustering and class analysis followed by scrupulous identification of social factors that could guide a Multi-Disciplinary Team (MDT) in personal social-occupational-ergonomics coaching, the objective being to provide an “Adapted Professional Activity” option [45].

Average length of the pathway to initial MDT pain evaluation exceeds 12 years of evolution for post-op chronic refractory back and/or leg pain [46]. Given the complexity of pathways and the impossibility of comparing, one by one, the options likely to yield the best outcomes for different subgroups of patients, one alternative solution would be to conduct large cohort prospective studies, where the primary endpoint would be not VAS decrease but rather a composite index aimed at reflecting global quality of life and taking account the different dimensions of pain. This would be a fertile substrate for further research perspectives based on the MCRI. 

### 4.3. The Need for a Polaroid Picture of Pain “In Color”, Required to Design a Novel Multi-Dimensional Composite Pain Assessment Index

As regards their overall profile, PSPS-T2 patients represent a vulnerable population, with limited capabilities for developing coping strategies and complex cognitive task elaboration processes. Rather than multiple independent questionnaires, they might benefit from a straightforward pain assessment, with reliable objective information, collected in a short amount of time, such as an MCRI composite index. 

In a recent topical review, Gewandter et al. [47] indicated that the main potential advantage of composite outcome measures is that they provide comprehensive assessments of complex pain. They highlighted the need to include clinical input with data from patients so as to ensure the clinical relevance of the composite score. Based on three different approaches, the author reported examples of published composite outcomes for pain. All the composite scores were built by combining cut-offs related to different scores [25]. For instance, Patel et al. [48] integrated input from NPRS and physical function (subscale of Short Form-36) to test 10 composite scores in 2287 painful diabetic peripheral neuropathy patients and 1513 postherpetic neuralgia patients, providing a composite score consisting of ≥50% improvement in pain or ≥20% improvement in pain combined with ≥30% improvement in physical function. Likewise, Pilitsis et al. [49], using data from 175 PSPS-T2 patients implanted with a spinal cord stimulation device, proposed an algorithmic composite score based on pain intensity, catastrophizing, quality of life and physical capability, the objective being to identify potential responders to spinal cord stimulation. The authors reported an average responder rate of 83.7% and 83.6% at 6- and 12-months respectively. The responder algorithm showed high agreement with PGIC (96%). While our own composite MCRI has shown lower sensitivity (77.4%) and specificity (79.8%), given the differences in therapies proposed (spinal cord stimulation vs. real-life medical management in PREDIBACK). it might be hazardous to transpose the results obtained by Pilitsis et al. [49] to a practical approach such as ours. Furthermore, it appears that responder rates and correlations to PGIC were obtained with an algorithm, which had not been compared to other scores, making it difficult to put the results in perspective with ours. By using machine learning, we have accurately determined the load of each item, rather than each outcome, showing that MCRI was more sensitive and specific with regard to PGIC compared to all other outcomes (ODI, EQ-5D, NRPS, HADS, PMI). Furthermore, our study provides a Minimum Clinically Important Difference (MICD) of 1.05 points, which can, through follow-up visits, detect pain changes with higher accuracy than other evaluation methods, [50]. In pain studies, a threshold of 30% or 2 points in VAS change [39] generally signals a significant difference between treatments. In our work, with MICD of 1.05/10 due to MCRI’s power of detection, we could exponentially increase the granularity of patient analysis and clustering. 

In conclusion, it appears safe to assume that the MCRI score offers new perspectives to delineate comprehensive relevant clinical approaches for PSPS-T2 patients. However, we are aware that composite measurements should not replace the individual domains of composite outcome analysis; indeed, there exists strong complementarity between the different ways of assessing, all of them aimed at determining the optimal individual pathway for a specific patient.

### 4.4. A Dynamic Multiplexed Vision of the Patient Pathway Focusing on Clinical Outcomes, Therapeutical Strategy Efficacy, Patient Profiling and AI-Based Outcome Predictions

Recently, Gewandter et al. [47] claimed that composite score can incorporate domains relevant to assessment of therapy efficacy. Following from that, MCRI would make it possible not only to assess therapeutical strategy efficacy with objective and robust metrics through complex pathways, but ultimately to provide a quantitative substrate to further medico-economic extrapolations and AI-based predictive medicine, which will delineate future indications, reimbursements, and optimized care by means of increasingly personalized therapy.

### 4.5. Strengths and Limitations of the Study

Even though our study is the first to develop a composite score of pain assessment, using a machine learning approach and through a prospective real-life study, in the general context of PSPS-T2 pain management substantial limitations need to be addressed. 

First, our current MCRI is specifically dedicated to PSPS-T2 patients and cannot be directly adapted to other pathologies or used to assess all therapy effects. While this first step constitutes a strong baseline for future studies, clinical validations on cohorts would be needed prior to initiation of a second phase. This would be aimed at stratifying therapeutical choices and at rationalizing patient pathways. 

Secondly, we have used “only” 5 dimensions to design the MCRI and are convinced that incorporation of other dimensions, such as a social component or Quality of Sleep, might reinforce the robustness of our model. In clinical practice, however, too many assessments might decrease individuals’ willingness to respond adequately to each questionnaire. This could impede enrolment and follow-up, especially in research, potentially compromising the quality of the collected source information. MCRI potential users will need to agree on the most acceptable compromise to address this index to vulnerable patients.

## 5. Conclusions

Because pain is a physical sensation integrating psychological and functional dimensions, assessment justifies the use of multi-dimensional tools such as composite indexes. Application of machine learning algorithms to pain intensity, pain surface, functional disability, psychological distress and quality of life led us to develop a novel Multidimensional Clinical Response Index (MCRI) to determine a composite pain score to accurately assess PSPS-T2 patients. MCRI appeared to be the best compromise among all existing indexes, showing the highest sensitivity/specificity related to Patient Global Impression of Change (PGIC). This approach can be considered as a launching pad to the development of further models designed to prospectively evaluate therapy effects, using robust tools.

## Figures and Tables

**Table 1:** Factor loadings and correlations between EFA-based factors.

Item	Functional Disability	Depression/Anxiety
EQ-5D_Mobility	0.53	0.05
EQ-5D_Self care	0.48	−0.07
EQ-5D_Usual activities	0.48	0.09
ODI_Personal care	0.46	0.03
ODI_Lifting	0.31	−0.07
ODI_Walking	0.47	−0.06
ODI_Sitting	0.54	−0.12
ODI_Standing	0.59	−0.12
ODI_Sleeping	0.32	0.11
ODI_Sex life	0.40	0.14
ODI_Social life	0.34	0.19
ODI_Travelling	0.47	0.05
HAD_Feeling tense or ‘wound up’	0.11	0.31
HAD_Enjoying things you used to enjoy	0.16	0.43
HAD_Feeling that something awful is about to happen	−0.10	0.52
HAD_Laughing and seeing the good side of things	0.04	0.50
HAD_Worrying thoughts going through your mind	−0.03	0.51
HAD_Feeling cheerful	0.05	0.45
HAD_Feeling slowed down	0.30	0.29
HAD_Frightened feeling like ‘butterflies’ in the stomach	−0.15	0.70
HAD_Looking forward with enjoyment to things	0.05	0.49
HAD_Sudden feeling of panic	−0.06	0.56
HAD_Enjoying a good book or radio/TV program	−0.09	0.40
Functional Disability	-	0.54

EQ-5D: EuroQol-5 Dimensions; ODI: Oswestry Disability Index; HAD: Hospital Anxiety and Depression.

**Table 2:** Multilevel CFA model results: Standardized coefficients, their 95% CIs and their significance *p*-values.

Latent Variables	Standardized Coefficients	95% CI	*p*-Value
Level 1: within-patients effects			
Functional Disability			
EQ5D_Mobility	1	-	-
EQ5D_Self care	0.555	[0.303,0.807]	<0.0001
EQ5D_Usual activities	0.971	[0.741,1.201]	<0.0001
ODI_Personal care	0.901	[0.494,1.307]	<0.0001
ODI_Lifting	0.663	[0.338,0.988]	<0.0001
ODI_Walking	0.703	[0.469,0.936]	<0.0001
ODI_Sitting	0.720	[0.492,0.947]	<0.0001
ODI_Standing	0.842	[0.582,1.102]	<0.0001
ODI_Sleeping	0.828	[0.488,1.168]	<0.0001
ODI_Sex life	1.231	[0.757,1.705]	<0.0001
ODI_Social life	1.354	[0.761,1.947]	<0.0001
ODI_Travelling	0.951	[0.607,1.295]	<0.0001
HAD_Feeling slowed down	0.944	[0.632,1.257]	<0.0001
Depression & anxiety			
HAD_Feeling tense or ’wound up’	1	-	-
HAD_Enjoying thing you used to enjoy	1.914	[1.231,2.597]	<0.0001
HAD_Feeling that something awful is about to happen	1.140	[0.492,1.789]	0.0006
HAD_Laughing and seeing the good side of things	1.560	[0.960,2.160]	<0.0001
HAD_Worrying thoughts going through your mind	1.094	[0.680,1.508]	<0.0001
HAD_Feeling cheerful	1.183	[0.802,1.563]	<0.0001
HAD_Frightened feeling like ’butterflies’ in the stomach	1.279	[0.765,1.793]	<0.0001
HAD_Looking forward with enjoyment to things	1.609	[1.108,2.110]	<0.0001
HAD_Sudden feeling of panic	1.003	[0.549,1.455]	<0.0001
HAD_Enjoying a good book or radio/TV program	0.822	[0.454,1.191]	<0.0001
Level 2: between-patients effects			
Functional Disability			
EQ5D_Mobility	1	-	-
EQ5D_Self care	0.928	[0.673,1.183]	<0.0001
EQ5D_Usual activities	0.840	[0.678,1.002]	<0.0001
ODI_Personal care	0.987	[0.683,1.291]	<0.0001
ODI_Lifting	0.878	[0.607,1.148]	<0.0001
ODI_Walking	1.041	[0.840,1.242]	<0.0001
ODI_Sitting	0.642	[0.373,0.911]	<0.0001
ODI_Standing	0.829	[0.607,1.051]	<0.0001
ODI_Sleeping	0.501	[0.288,0.713]	<0.0001
ODI_Sex life	0.865	[0.427,1.304]	0.00011
ODI_Social life	0.901	[0.534,1.267]	<0.0001
ODI_Travelling	0.748	[0.555,0.941]	<0.0001
HAD_Feeling slowed down	0.434	[0.227,0.641]	<0.0001
Depression & anxiety			
HAD_Feeling tense or ’wound up’	1	-	-
HAD_Enjoying thing you used to enjoy	0.857	[0.216,1.496]	0.0088
HAD_Feeling that something awful is about to happen	1.740	[1.160,2.320]	<0.0001
HAD_Laughing and seeing the good side of things	1.078	[0.572,1.583]	<0.0001
HAD_Worrying thoughts going through your mind	1.482	[1.130,1.834]	<0.0001
HAD_Feeling cheerful	0.820	[0.412,1.228]	<0.0001
HAD_Frightened feeling like ’butterflies’ in the stomach	1.393	[0.860,1.925]	<0.0001
HAD_Looking forward with enjoyment to things	0.819	[0.219,1.419]	0.0075
HAD_Sudden feeling of panic	1.392	[0.784,2.001]	<0.0001
HAD_Enjoying a good book or radio/TV program	0.339	[−0.009,0.687]	0.057

EQ-5D: EuroQol-5 Dimensions; ODI: Oswestry Disability Index; NPRS: Numeric Pain Rating Scale; HADS: Hospital Anxiety and Depression.

**Table 3:** Goodness of fit measures for the CFA model obtained by including the 5 factors obtained from EFA and the CFA models including each factor and its associated items.

Goodness of Fit Measures	Two Factor CFA Model	Functional Disability	Depression & Anxiety
Chi-squared test *	Chi2 = 940.66; *p* < 0.0001	Chi2 = 333.84; *p* < 0.0001	Chi2 = 260.00; *p* < 0.0001
RMSAE	0.046; 90% CI = [0.042,0.050]	0.056; 90% CI = [0.048,0.063]	0.064; 90% CI = [0.056,0.072]
Robust CFI	0.848	0.876	0.886

* with the Yuan–Bentler correction.

**Table 4:** Correlations at baseline between the scores obtained from EFA and the standard pain evaluation measures.

	ODI Score	EQ-5D Index	NPRS	HADS Total Score	Mapping Intensity
Baseline					
Functional Disability	0.92 95% CI = [0.89,0.94]	−0.81 95% CI = [−0.86,−0.74]	0.40 95% CI = [0.24,0.53]	0.35 95% CI = [0.20,0.49]	0.30 95% CI = [0.14,0.44]
Depression/Anxiety	0.38 95% CI = [0.23,0.52]	−0.49 95% CI = [−0.60,−0.35]	0.35 95% CI = [0.20,0.49]	0.96 95% CI = [0.94,0.97]	−0.02 95% CI = [−0.19,0.15]
M3					
Functional Disability	0.95 95% CI = [0.93,0.97]	−0.83 95% CI = [−0.88,−0.75]	0.52 95% CI = [0.36,0.65]	0.48 95% CI = [0.32,0.61]	0.30 95% CI = [0.12,0.47]
Depression/Anxiety	0.51 95% CI = [0.34,0.64]	−0.53 95% CI = [−0.65,−0.37]	0.36 95% CI = [0.18,0.52]	0.97 95% CI = [0.96,0.98]	0.04 95% CI = [−0.15,0.23]
M6					
Functional Disability	0.97 95% CI = [0.96,0.98]	−0.87 95% CI = [−0.91,−0.81]	0.68 95% CI = [0.55,0.78]	0.53 95% CI = [0.37,0.66]	0.26 95% CI = [0.07,0.43]
Depression/Anxiety	0.56 95% CI = [0.41,0.68]	−0.62 95% CI = [−0.73,−0.48]	0.48 95% CI = [0.31,0.62]	0.98 95% CI = [0.96,0.98]	0.11 95% CI = [−0.09,0.30]
M9					
Functional Disability	0.96 95% CI = [0.95,0.98]	−0.87 95% CI = [−0.91,−0.80]	0.60 95% CI = [0.44,0.72]	0.48 95% CI = [0.30,0.63]	0.36 95% CI = [0.16,0.53]
Depression/Anxiety	0.55 95% CI = [0.39,0.68]	−0.60 95% CI = [−0.71,−0.44]	0.41 95% CI = [0.21,0.57]	0.97 95% CI = [0.96,0.98]	0.24 95% CI = [0.03,0.43]
M12					
Functional Disability	0.96 95% CI = [0.95,0.98]	−0.86 95% CI = [−0.91,−0.79]	0.59 95% CI = [0.43,0.71]	0.57 95% CI = [0.40,0.70]	0.38 95% CI = [0.18,0.55]
Depression/Anxiety	0.54 95% CI = [0.38,0.68]	−0.52 95% CI = [−0.66,−0.35]	0.40 95% CI = [0.20,0.56]	0.92 95% CI = [0.88,0.95]	0.07 95% CI = [−0.15,0.28]

EQ-5D: EuroQol-5 Dimensions; ODI: Oswestry Disability Index; NPRS: Numeric Pain Rating Scale; HADS: Hospital Anxiety and Depression Scale.

**Table 5:** Composition of the first principal component.

Variables	1st Principal Component: 49.99% of the Total Variance
Eigenvalue	1.99
Functional Disability score	−0.811
NPRS score	−0.771
Depression & anxiety score	−0.684
Mapping intensity score	−0.529

NPRS: Numeric Pain Rating Scale.

**Table 6:** Correlation matrix of the MCRI, ODI score, EQ-5D index, total HADS score and mapping intensity at M0, M3, M6, M9 and M12 follow-ups.

Variables	ODI	EQ-5D	NPRS	Total HADS	Mapping Intensity
Correlations at baseline					
MCRI	−0.677 ***	0.690 ***	−0.677 ***	−0.622 ***	−0.423 ***
ODI	1	−0.631 ***	0.415 ***	0.279 ***	0.272 ***
EQ-5D	-	1	−0.346 ***	−0.434 ***	−0.250 ***
NPRS	-	-	1	0.289 ***	0.235 **
Total HADS	-	-	-	1	0.131
Mapping intensity	-	-	-	-	1
Correlations at 3 months					
MCRI	−0.771 ***	0.714 ***	−0.761 ***	−0.616 ***	−0.527 ***
ODI	1	−0.728 ***	0.567 ***	0.472 ***	0.261 ***
EQ-5D	-	1	−0.521 ***	−0.492 ***	−0.244 **
NPRS	-	-	1	0.406 ***	0.322 ***
Total HADS	-	-	-	1	0.050
Mapping intensity	-	-	-	-	1
Correlations at 6 months					
MCRI	−0.762 ***	0.788 ***	−0.828 ***	−0.631 ***	−0.537 ***
ODI	1	−0.776 ***	0.689 ***	0.542 ***	0.262 ***
EQ-5D	-	1	−0.659 ***	−0.586 ***	−0.310 ***
NPRS	-	-	1	0.442 ***	0.455 ***
Total HADS	-	-	-	1	0.106
Mapping intensity	-	-	-	-	1
Correlations at 9 months					
MCRI	−0.775 ***	0.803 ***	−0.774 ***	−0.655 ***	−0.637 ***
ODI	1	−0.784 ***	0.608 ***	0.611 ***	0.314 ***
EQ-5D	-	1	−0.645 ***	−0.581 ***	−0.350 ***
NPRS	-	-	1	0.483 ***	0.424 ***
Total HADS	-	-	-	1	0.244 **
Mapping intensity	-	-	-	-	1
Correlations at 12 months					
MCRI	−0.758 ***	0.730 ***	−0.806 ***	−0.603 ***	−0.638 ***
ODI	1	−0.749 ***	0.514 ***	0.476 ***	0.456 ***
EQ-5D	-	1	−0.544 ***	−0.520 ***	−0.489 ***
NPRS	-	-	1	0.376 ***	0.553 ***
Total HADS	-	-	-	1	0.291 ***
Mapping intensity	-	-	-	-	1

** *p* < 0.01; *** *p* < 0.001. MCRI: Multidimensional Clinical Response Index; EQ-5D: EuroQol-5 Dimensions; ODI: Oswestry Disability Index; NPRS: Numeric Pain Rating Scale; HADS: Hospital Anxiety and Depression.

**Table 7:** The specificity and sensitivity of MCRI, ODI, EQ-5D, NPRS, HADS and mapping intensity at detecting patient satisfaction with their perceived change at each visit. To allow comparability, cutoffs maximizing specificity and sensitivity were identified for each score.

	Satisfactory Self-Perceived Clinical Improvement	Unsatisfactory Self-Perceived Clinical Improvement
Change between M0–M3		
MCRI ≥ 1.05 < 1.05	16 (sensitivity = 64.0%) 9	29 95 (specificity = 76.6%)
ODI ≥ 6.7 < 6.7	14 (sensitivity = 56.0%) 11	27 93 (specificity = 77.5%)
EQ-5D ≥ 0.13 < 0.13	12 (sensitivity = 50.0%) 12	38 79 (specificity = 67.5%)
NPRS ≥ 2 < 2	13 (sensitivity = 52.0%) 12	25 88 (specificity = 77.9%)
HADS ≥ 5 < 5	14 (sensitivity = 56.0%) 11	23 97 (specificity = 80.8%)
Mapping intensity≥ 468 < 468	15 (sensitivity = 60.0%) 10	44 77 (specificity = 63.6%)
Change between M0–M6		
MCRI ≥ 1.05 < 1.05	19 (sensitivity = 65.5%) 10	25 90 (specificity = 78.3%)
ODI ≥ 6.7 < 6.7	21 (sensitivity = 72.4%) 8	33 78 (specificity = 70.3%)
EQ-5D ≥ 0.13 < 0.13	21 (sensitivity = 75.0%) 7	31 72 (specificity = 69.9%)
NPRS ≥ 2 < 2	19 (sensitivity = 70.4%) 8	28 72 (specificity = 72.0%)
HADS ≥ 5 < 5	17 (sensitivity = 58.6%) 12	21 90 (specificity = 81.1%)
Mapping intensity ≥ 468 < 468	19 (sensitivity = 65.5%) 10	51 61 (specificity = 54.5%)
Change between M0–M9		
MCRI ≥ 1.05 < 1.05	22 (sensitivity = 75.9%) 7	22 77 (specificity = 77.8%)
ODI ≥ 6.7 < 6.7	19 (sensitivity = 65.5%) 10	27 71 (specificity = 72.4%)
EQ-5D ≥ 0.13 < 0.13	16 (sensitivity = 57.1%)12	30 65 (specificity = 68.4%)
NPRS ≥ 2 < 2	20 (sensitivity = 69.0%)9	33 60 (specificity = 64.5%)
HADS ≥ 5 < 5	17 (sensitivity = 58.6%) 12	20 78 (specificity = 79.6%)
Mapping intensity ≥ 468 < 468	12 (sensitivity = 55.6%) 15	37 57 (specificity = 60.6%)
Change between M0–M12		
MCRI ≥ 1.05 < 1.05	24 (sensitivity = 77.4%) 7	19 75 (specificity = 79.8%)
ODI ≥ 6.7 < 6.7	20 (sensitivity = 64.5%) 11	27 66 (specificity = 71.0%)
EQ-5D ≥ 0.13 < 0.13	20 (sensitivity = 69.0%) 9	29 62 (specificity = 68.1%)
NPRS ≥ 2 < 2	22 (sensitivity = 71.0%) 9	27 59 (specificity = 68.6%)
HADS ≥ 5 < 5	20 (sensitivity = 64.5%) 11	22 71 (specificity = 76.3%)
Mapping intensity≥ 468 < 468	19 (sensitivity = 65.5%) 10	31 59 (specificity = 65.6%)

MCRI: Multidimensional Clinical Response Index; EQ-5D: EuroQol-5 Dimensions; ODI: Oswestry Disability Index; NPRS: Numeric Pain Rating Scale; HADS: Hospital Anxiety and Depression.

## Data Availability

Not applicable.
